# Cytogenetic study of heptapterids (Teleostei, Siluriformes) with particular respect to the *Nemuroglanis* subclade

**DOI:** 10.3897/CompCytogen.v9i1.8488

**Published:** 2015-02-05

**Authors:** Daniel Luis Zanella Kantek, Wellington Adriano Moreira Peres, Orlando Moreira-Filho

**Affiliations:** 1 Taiamã Ecological Station, Chico Mendes Biodiversity Conservation Institute, Mato Grosso, Brazil; 2 Environmental Protection Area Meanders of the Araguaia river, Chico Mendes Biodiversity Conservation Institute, Mato Grosso, Brazil; 3 Laboratory of Molecular Biodiversity and Cytogenetics, Department of Genetics and Evolution, Federal University of São Carlos, São Paulo, Brazil

**Keywords:** Siluriformes, Heptapteridae, chromosomes, 5S and 18S rDNA, cytotaxonomy

## Abstract

The catfish family Heptapteridae (order Siluriformes) is endemic to the Neotropics and is one of the most common of the fish families in small bodies of water. Although over 200 species have been identified in this family, very few have been characterized cytogenetically. Here, we analyze the chromosome genomes of four species of Heptapteridae: *Cetopsorhamdia iheringi* (Schubart & Gomes, 1959), 2n = 58, comprising 28 metacentric (m) + 26 submetacentric (sm) + 4 subtelomeric (st) chromosomes; *Pimelodella vittata* (Lütken, 1874), 2n = 46, comprising 16m + 22sm + 8st; Rhamdia prope quelen (Quoy & Gaimard, 1824), 2n = 58 comprising 26m + 16sm + 14st + 2 acrocentric; and Rhamdiopsis prope microcephala (Lütken, 1874), 2n = 56, comprising 12m + 30sm + 14st. The nucleolus organizer regions (NORs) were located in a single chromosome pair in all species. The two species that belonged to the subclade *Nemuroglanis*, *Cetopsorhamdia iheringi* and Rhamdia prope quelen, had a diploid chromosome number of 58 and an interstitial NOR adjacent to a C^+^ block located on one of the larger chromosome pairs in the complement. Our results from conventional cytogenetic techniques in combination with FISH using 18S and 5S rDNA probes corroborated the taxonomical hypothesis for the formation of the *Nemuroglanis* subclade.

## Introduction

In recent years, various classification changes have led to the current taxonomic status of the catfish family Heptapteridae. Lundberg et al. (1991a, b) suggested the division of the family Pimelodidae into the subfamilies Pimelodinae, Pseudopimelodinae, and Rhamdiinae. Subsequently, on the basis of phylogenetic studies in the Siluriformes, Pinna (1998) elevated the subfamily Rhamdiinae to the level of a family, Rhamdiidae. Bockmann and Guazzelli (2003) later established the family Heptapteridae instead of Rhamdiidae; this family includes 24 genera and 189 valid species (Ferraris 2007) of small fish, commonly known as “bagres” or “mandis”. These fish are characterized by a long adipose fin, three pairs of barbels, an elongated body, and a grayish body color. They are endemic to the Neotropics and have a wide distribution in the water courses of Central and South America, with many species distributed in areas of ichthyological endemism. They live on the bottom of small and medium rivers, at low to medium depths, and are usually solitary with nocturnal habits (Bockmann and Guazelli 2003).

Subclades of Rhamdiinae (= Heptapteridae) have been identified in phylogenetic analyses of morphological data (Ferraris 1988, Lundberg et al. 1991a, Bockmann 1994): *Rhamdia* (Bleeker, 1858) and *Pimelodella* (Eigenmann & Eigenmann, 1888) are assigned to a basal group; while *Cetopsorhamdia* (Eigenmann & Fisher, 1916) and *Rhamdiopsis* (Haseman, 1911) have been placed in the *Nemuroglanis* subclade.

The diploid chromosome number in the Heptapteridae varies from 2n = 42 in *Imparfinis hollandi* (Haseman, 1911) (Margarido and Moreira-Filho 2008) to 2n = 58 in many other species. The latter chromosome number is the most frequent and is also considered a plesiomorphic character (Swarça et al. 2007, Borba et al. 2011). The karyotypes of heptapterid species comprise mainly metacentric and submetacentric chromosomes (see below) suggesting that pericentric inversions were more frequent than centric fissions in the evolution of the family. Nucleolus organizer regions (NORs) are usually present on one chromosome pair, and may be terminal or interstitial. These data suggest that extensive chromosomal rearrangements were involved in speciation within this group (Swarca et al. 2007). The reduction in diploid number may have been produced by successive chromosome fusions with deletions and inversions, such as those responsible for NOR position variation among species. B chromosomes are present in some species and are considered to be of recent origin, and without phylogenetic implications (Borba et al. 2011).

The presence of an interstitial NOR, which is usually located on the largest chromosome pair of the complement and adjacent to a C^+^ block, and the predominance of 2n = 58, are all cytogenetic characters strongly associated with the *Nemuroglanis* subclade (Kantek et al. 2009).

As there have been relatively few cytogenetic studies in the Heptapteridae, and because of the need to obtain further data to substantiate proposals on the cytotaxonomy of the family (Borba et al. 2011), the present study was undertaken to provide the first analysis, to our knowledge, of the karyotype of *Pimelodella vittata* (Lütken, 1874). We also used various cytological methods to analyze three other heptapterid species and compared the new data with those previously published to examine the cytotaxonomy of this family.

## Material and methods

Specimens of four heptapterid species were collected from the Minhocas stream (S20°31'55.2", W046°02'42.1"), a tributary of the Piumhi river (Minas Gerais state): nine (seven males and two females) *Cetopsorhamdia iheringi* (Schubart & Gomes, 1959) (MNRJ 31477); six (five males and one female) *Pimelodella vittata* (MNRJ 29330); 10 (five males, four females and one of an undetermined sex) Rhamdia prope quelen (Quoy & Gaimard, 1824) (MNRJ 29329, MNRJ 29326); and 18 (eight males, seven females and three of undetermined sex) of Rhamdiopsis prope microcephala (Lütken, 1874) (MNRJ 29325).

Mitotic metaphase preparations were made as described by Bertollo et al. (1978). Chromosome morphologies were assigned using the arm size ratio criteria proposed by Levan et al. (1964). Heterochromatin was identified by C-banding (Sumner 1972) and NORs were detected by silver nitrate staining (Howell and Black 1980). Metaphase preparations analyzed after conventional staining (Giemsa) were also subjected to C-banding, allowing the assemblage of sequential karyotypes.

The 18S and 5S rDNA sites on the chromosomes were located by the fluorescence *in situ* hybridization (FISH) technique (Pinkel et al. 1986), with a stringency of 77%, using probes obtained from *Prochilodus argenteus* (Agassis, 1829) (Hatanaka and Galetti Jr 2004) and *Leporinus elongatus* (Valenciennes, 1850) (Martins and Galetti Jr 2001), respectively. The two probes were labeled with 14-dATP-biotin through nick translation in accordance with the manufacturer’s instructions (Bionick Labelling System, Invitrogen). Chromosomes were counterstained with DAPI (0.2 mg/ml) and analyzed using an Olympus BX50 epifluorescence microscope. Image-Pro Plus software (Media Cybernetics) was used for image capture.

## Results

### Cetopsorhamdia iheringi

**Figure 1.**
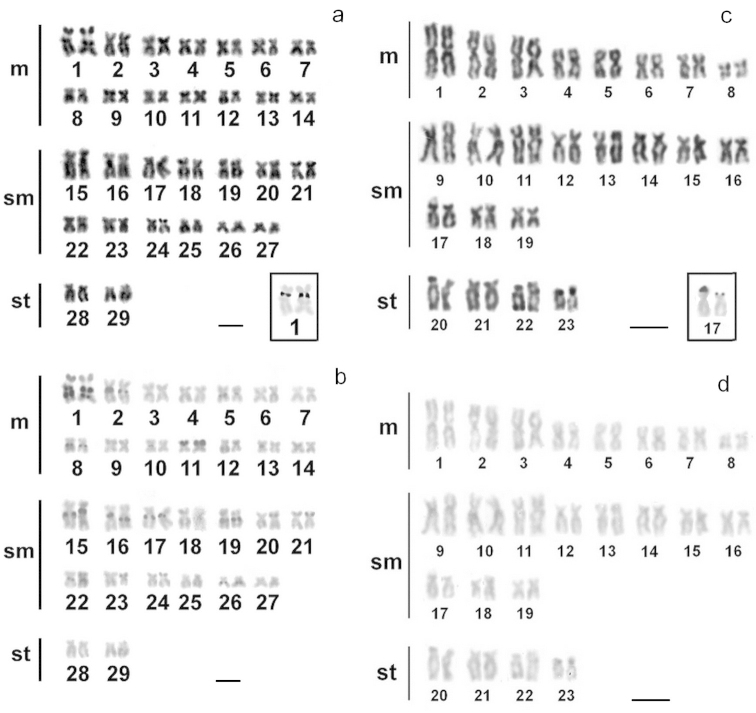
Karyotypes of *Cetopsorhamdia iheringi* (**a, b**) and *Pimelodella vittata* (**c, d**) after sequential Giemsa staining (**a, c**), C- banding (**b, d**) and Ag-NOR staining (boxes). Bar = 10 µm.

Cells from all *Cetopsorhamdia iheringi* specimens had 2n = 58 and a karyotypic formula of 28 metacentric (m), 26 submetacentric (sm) and 4 subtelocentric (st) chromosomes (Fig. 1a), with no evidence of heteromorphic sex chromosomes.

Silver staining showed that the NOR was located interstitially on the short arm of pair 1, and formed a secondary constriction (Fig. 1a box). Constitutive heterochromatin was present in the pericentromeric regions of several chromosome pairs (Fig. 1b) in addition to visible C^+^ blocks in the NOR-bearing pair (Fig. 1a, b).

FISH with the 18S rDNA probe confirmed that the NOR was located interstitially on the short arm of pair 1 (Fig. 4a). FISH using the 5S ribosomal probe revealed the existence of a large number of these sequences on the NOR-bearing chromosomes, covering a large part of the chromosomes above and below the 18S ribosomal sites. There was synteny between the 18S and 5S rDNAs (Fig. 4b, c).

### Pimelodella vittata

All cells from *Pimelodella vittata* specimens had 2n = 46 and a karyotypic formula of 16m, 22sm and 8st chromosomes (Fig. 1c), with no evidence of heteromorphic sex chromosomes.

Silver staining located the NORs to the terminal region of the short arm of pair 17, where they formed a secondary constriction (Fig. 1c box). It was possible to see weak C^+^ bands close to the centromeres in some chromosomes (Fig. 1d).

FISH using the 18S rDNA probe confirmed the NOR location (Fig. 4d). Only one 5S rDNA locus was present in *Pimelodella vittata* in the terminal region of a submetacentric/subtelocentric chromosome pair (Fig. 4e). The 18S and 5S rDNA loci were not on the same pair of chromosomes (Fig. 4e, f).

### Rhamdia prope quelen

**Figure 2.**
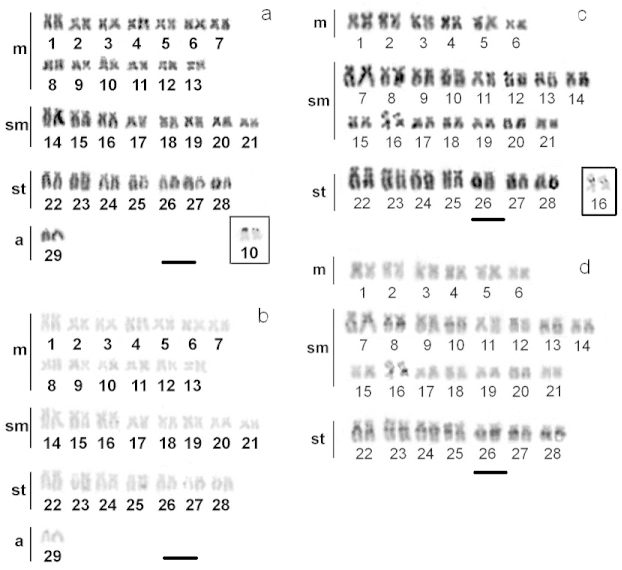
Karyotypes of Rhamdia prope quelen (**a, b**) and Rhamdiopsis prope microcephala (**c, d**) after sequential Giemsa staining (**a, c**), C- banding (**b, d**) and Ag-NOR staining (boxes). Bar = 10 µm.

**Figure 3.**
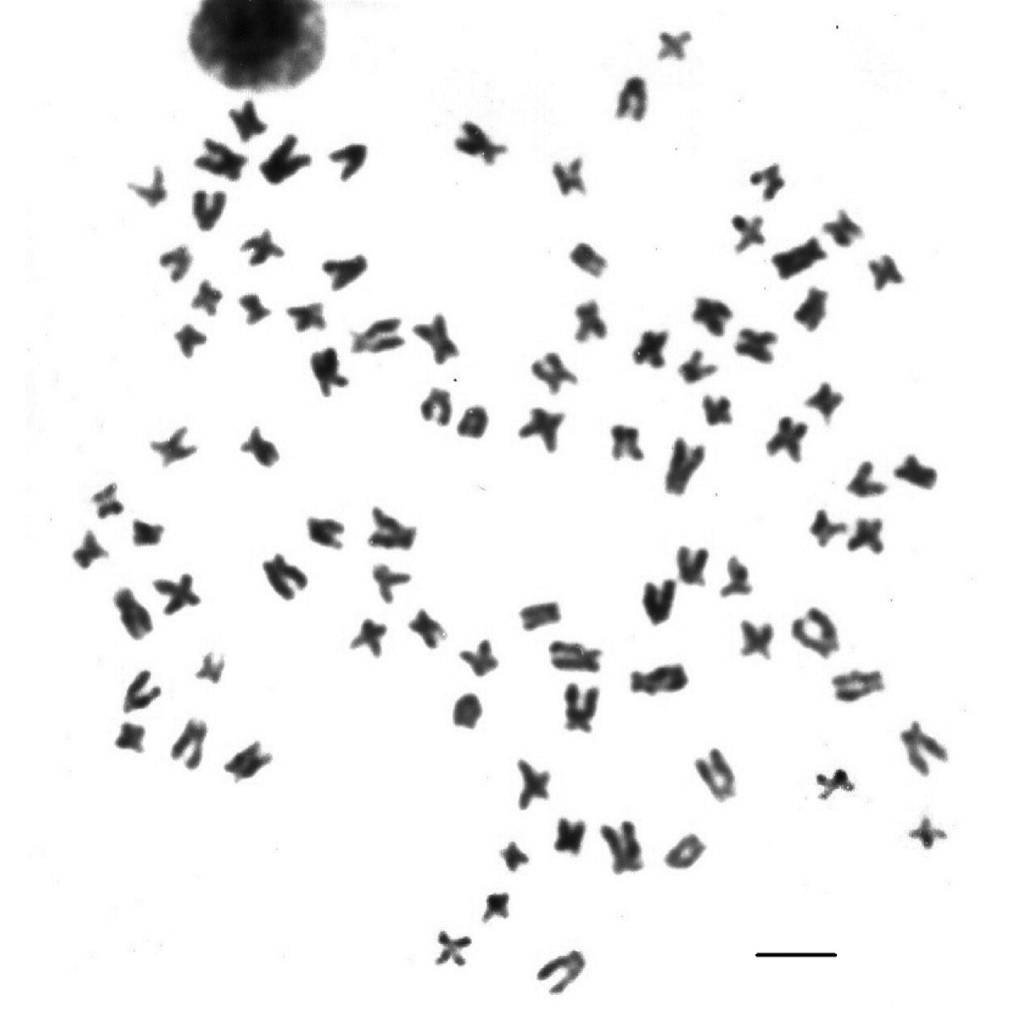
Metaphase of the triploid specimen of Rhamdia prope quelen. Bar = 10 µm.

Cells from all specimens, apart from one, had 2n = 58 and a karyotypic formula of 26m, 16sm, 14st and 2 acrocentric chromosomes (Fig. 2a), with no evidence of heteromorphic sex chromosomes. One triploid specimen with 3n = 87 was found (Fig. 3).

Silver staining indicated the NOR was located in the terminal region of chromosome pair 10, where it formed a secondary constriction (Fig. 2a box). The chromosomes did not show any heterochromatic segments (Fig. 2b).

FISH using the 18S rDNA probe hybridized to the same region as the Ag-NOR (Fig. 5a, c). Only one 5S rDNA locus was identified; this was located at an interstitial position on a submetacentric chromosome pair (Fig. 5b, d).

**Figure 4.**
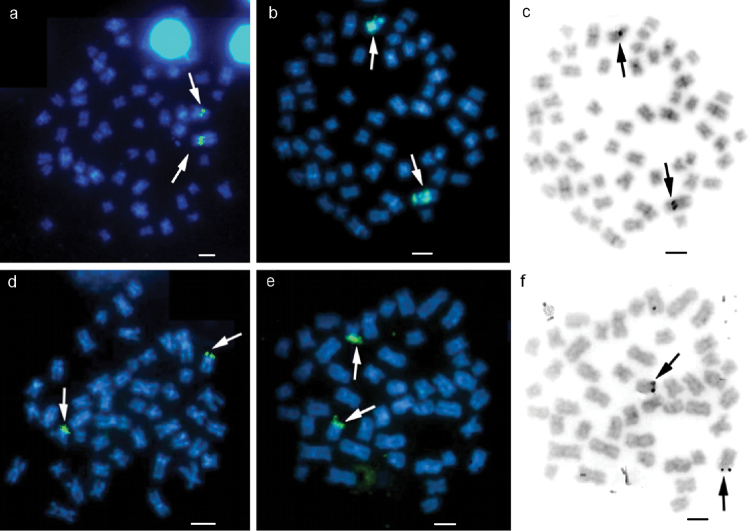
Metaphases of *Cetopsorhamdia iheringi* (**a, b, c**) and *Pimelodella vittata* (**d, e, f**) subjected to fluorescence *in situ* hybridization (FISH) with an 18S rDNA probe (**a, d**) and 5S rDNA (**b, e**). The metaphases shown after Ag-NOR staining (**c, f**) are the same as those used for 5S FISH. Bar =10 µm.

**Figure 5.**
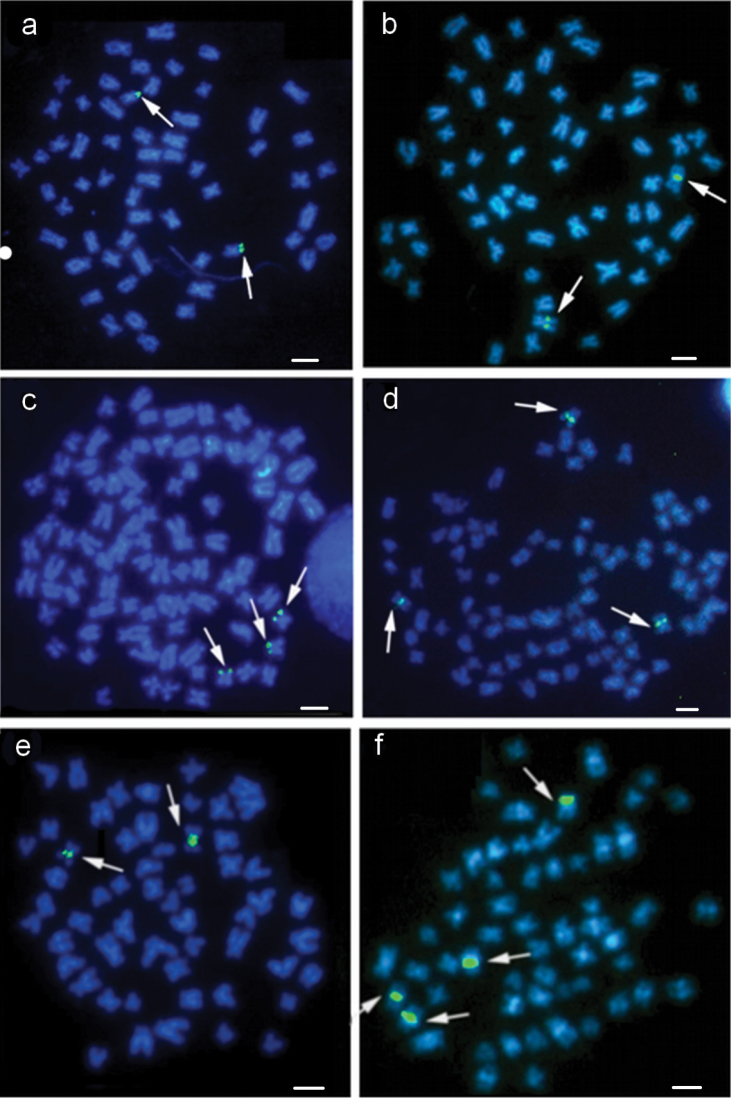
Metaphases of Rhamdia prope quelen (**a, b, c, d**) and Rhamdiopsis prope microcephala (**e, f**) subjected to fluorescence *in situ* hybridization with an 18S rDNA probe (**a, c, e**) and 5S rDNA (**b, d, f**). Metaphases c and d belong to the triploid specimen. Bar =10 µm.

### Rhamdiopsis prope microcephala

Cells from all specimens had 2n = 56 and a karyotypic formula of 12m, 30sm and 14st chromosomes (Fig. 2c), with no evidence of heteromorphic sex chromosomes.

Silver staining indicated the NOR was located in an interstitial region of chromosome pair 16, where it formed a secondary constriction (Fig. 2c box). Constitutive heterochromatin was present in the pericentromeric regions of several chromosome pairs (Fig. 2d).

FISH using the 18S rDNA probe hybridized to the same region as the Ag-NOR (Fig. 5e). Two 5S rDNA loci were identified at a terminal position on a submetacentric/subtelocentric chromosome pair (Fig. 5f).

## Discussion

The diploid chromosome number of 58 in *Cetopsorhamdia iheringi* and Rhamdia prope quelen is the most common karyotype number in the family Heptapteridae (Fenocchio and Bertollo 1990, Vissotto et al. 1999, Vissotto et al. 2001, Stolf et al. 2004, Kantek et al. 2009, Borba et al. 2011). The karyotype of 2n = 46 observed here in *Pimelodella vittata* is the same as reported for some other *Pimelodella* spp. (Dias and Foresti 1993, Vasconcelos and Martins-Santos 2000, Garcia and Almeida-Toledo 2010), *Pimelodella avanhandavae* (Eigenmann, 1917) (Vissoto et al. 1999), *Pimelodella meeki* (Eigenmann, 1910) (Vidotto et al. 2004, Garcia and Almeida-Toledo 2010, Borba et al. 2011, Gouveia et al. 2012), *Pimelodella boschmai* (Van der Stigchal, 1964) (Garcia and de Almeida-Toledo 2010) and *Pimelodella gracilis* (Valenciennes, 1836) (Garcia and de Almeida-Toledo 2010). Other *Pimelodella* species have different diploid chromosome numbers (Vasconcelos and Martins-Santos 2000, Swarça et al. 2003, Garcia and de Almeida-Toledo 2010).

The identification of a triploid specimen (3n = 87) in Rhamdia prope quelen is not unusual; indeed, three other cases have already been reported for *Rhamdia* (Swarça et al. 2007, Tsuda et al. 2010). The fertilization of a non-reduced (diploid) gamete by a reduced (haploid) gamete, such as an ovule (2n) by a sperm (n), is the most probable origin of these specimens (Morelli et al. 1983, Kantek et al. 2007).

The *Nemuroglanis* subclade is characterized by the presence of an interstitial NOR adjacent to a C^+^ block and the predominance of 2n = 58; these characteristics are present in the analyzed species from the genus *Cetopsorhamdia* (Vissoto et al. 1999 and present study), *Taunayia bifaciata* (Eigenmann & Norris, 1900) (Borba et al. 2011) and in five species of the genus *Imparfinis* (Eigenmann & Norris, 1900) (Kantek et al. 2009, Borba et al. 2011, Gouveia et al. 2012). If 2n = 58 is a plesiomorphic trait of the Heptapteridae family (Borba et al. 2011), then the reduction to 2n = 56 might indicate synapomorphy, grouping Imparfinis prope piperatus (Vissoto et al. 2001, Fenocchio et al. 2003), Rhamdiopsis prope microcephala (present study) and *Rhamdiopsis microcephala* (Lütken, 1874) (Fonseca et al. 2003). The hypothesis is supported by the presence of an interstitial NOR located on chromosomes that are not metacentric and not the largest in the karyotype of these species. The species *Phenacorhamdia tenebrosa* (Schubart, 1964), which belongs to the *Nemuroglanis* subclade, also has 2n = 58 (Borba et al. 2011), but no interstitial NOR. Since 2n = 58 is considered the basal number for Heptapteridae (Fenocchio et al. 2003, Borba et al. 2011), and the species *Imparfinis borodini* (Mees & Cala, 1989) (Vissoto et al. 1999), *Imparfinis hollandi* (Margarido and Moreira-Filho 2008) and *Heptapterus mustelinus* (Valenciennes, 1835) (Yano and Margarido 2012) have a reduced diploid number (2n = 52, 2n = 42 and 2n = 54, respectively), it is possible that Robertsonian translocations were responsible for the karyotypic changes.

The C-banding and Ag-NOR patterns of *Rhamdia* and *Pimelodella* species (Swarça et al. 2007, Borba et al. 2011, Gouveia et al. 2012) are distinctly different from most taxa of the *Nemuroglanis* subclade that have been analyzed. The existence of cytogenetic characteristics that separate the recognized groups of Heptapteridae was initially proposed by Fenocchio et al. (2003). Thus, for example, the interstitial C^+^ band pattern is a more common feature of species of the *Nemuroglanis* subclade, such as *Cetopsorhamdia iheringi* and Rhamdiopsis prope microcephala (Fig. 1b, 2d, respectively). Other species of the family Heptapteridae that do not belong to this subclade, such as *Pimelodella vittata* (Fig. 1d) and Rhamdia prope quelen (Fig. 2b), have different patterns of heterochromatin distribution (Swarça et al. 2007, Garcia et al. 2010, Garcia and Almeida-Toledo 2010). However, as the majority of heptapterid species have not been studied cytogenetically studied, then it is difficult to elaborate broader proposals.

Another cytogenetic characteristic that may be diagnostic of the *Nemuroglanis* subclade is the synteny between 18S and 5S rDNA. Up until now, only *Imparfinis schubarti* (Gomes, 1956) (Kantek et al. 2009) and *Cetopsorhamdia iheringi* (present study) have been found to show this characteristic. Other genera in the Heptapteridae that do not belong to the *Nemuroglanis* subclade, such as *Pimelodella* and *Rhamdia* (Garcia et al. 2003, Garcia et al. 2010, present study), do not show this synteny. However, as Rhamdiopsis prope microcephala did not have this characteristic, then the association between 5S and 18S rDNA might be a synapomorphy, shared by the group of species in the *Nemuroglanis* subclade that have a 2n = 58 karyotype and possess interstitial NORs on the largest chromosome pair of the complement.

The 5S ribosomal gene consists of multiple copies of a highly conserved 150 base pair sequence, separated by highly variable non-transcribed spacers (Williams and Strobeck 1985). These variable sequences, which were caused by insertions/deletions, mini-repetitions and pseudogenes, are useful for evolutionary studies and serve as population markers for many organisms, including plants (Zanke et al. 1995), mammals (Suzuki et al. 1994) and fishes (Martins et al. 2002). Variations in these spacers have also been detected in some neotropical fishes, such as *Leporinus* (Martins and Galetti Jr 2001) and *Brycon* (Wasko et al. 2001). A comparison of the outcome of analysis of *Imparfinis schubarti* (Kantek et al. 2009) and *Cetopsorhamdia iheringi* indicates that despite the relative evolutionary proximity of the species (both belong to the *Nemuroglanis* clade), and the likely localization of these sequences to homeologous chromosomes, there is nevertheless considerable differences in the signals obtained with the 5S rDNA probe. The large 5S rDNA blocks on *Cetopsorhamdia iheringi* chromosomes presumably originated through duplication of the 5S rDNA of an ancestral species close to these taxa. Other species of heptapterids considered more basal in the family, such as species belonging to the genera *Rhamdia* and *Pimelodella*, have only small 5S rDNA signals; this suggests that the presence of the large 5S rDNA block in *Cetopsorhamdia iheringi* is an apomorphic character. Based on the supposed homogeneity among 5S rDNA repeats, several studies have proposed that 5S rDNA is subject to concerted evolution (Arnheim 1983), where duplicated gene family members evolve as a single unit that undergoes a high degree of homogenization (as a unit in concert) (Pinhal et al. 2011).

Prior to this study, variability in the number and location of 5S ribosomal genes has been reported among Siluriformes (Kavalko et al. 2004) except for *Rhamdia* (Garcia et al. 2010). The analyses here confirm the variability observed by other authors, and also the conservation of 5S rDNA in *Rhamdia*.

Until now, only the genera *Imparfinis*, *Cetopsorhamdia*, *Heptapterus*, *Phenacorhamdia*, *Rhamdiopsis*, *Pimelodella*, *Rhamdia*, and *Taunayia* had been cytogenetically analyzed; these represent only eight of the 24 genera in the family Heptapteridae (Yano and Margarido 2012). The first five belong to the subclade *Nemuroglanis*. More studies involving this family may assist in the elucidation of cytotaxonomy and chromosome evolution in this family.
